# Forced migration as a risk factor for COVID-19 infection in Africa: insight from Agadez, Niger

**DOI:** 10.11604/pamj.2021.40.97.28116

**Published:** 2021-10-13

**Authors:** Aboubacar Abdou Batoure, Oumarou Batoure, Blanche-Philomene Melanga Anya, Didier Tambwe, Bienvenu Baruani, Ishag El Khalef, Joseph Nsiari-Muzeyi Biey, Patrick Katoto, Charles Shey Wiysonge

**Affiliations:** 1 World Health Organization, Niamey, Niger,; 2 World Health Organization, Ouagadougou, Burkina Faso,; 3 Centre for Infectious Diseases, Faculty of Medicine and Health Sciences, Stellenbosch University, Cape Town, South Africa,; 4 Centre for Tropical Diseases and Global Health, Faculty of Medicine, Catholic University of Bukavu, Bukavu, Democratic Republic of Congo,; 5 Department of Global Health, Faculty of Medicine and Health Sciences, Stellenbosch University, Cape Town, South Africa,; 6 Cochrane South Africa, South African Medical Research Council, Cape Town, South Africa,; 7 School of Public Health and Family Medicine, University of Cape Town, Cape Town, South Africa

**Keywords:** SARS-COV-2, humanitarian crisis, migrants, mobility

## To the editors of the Pan African Medical Journal

**Figure 1:**
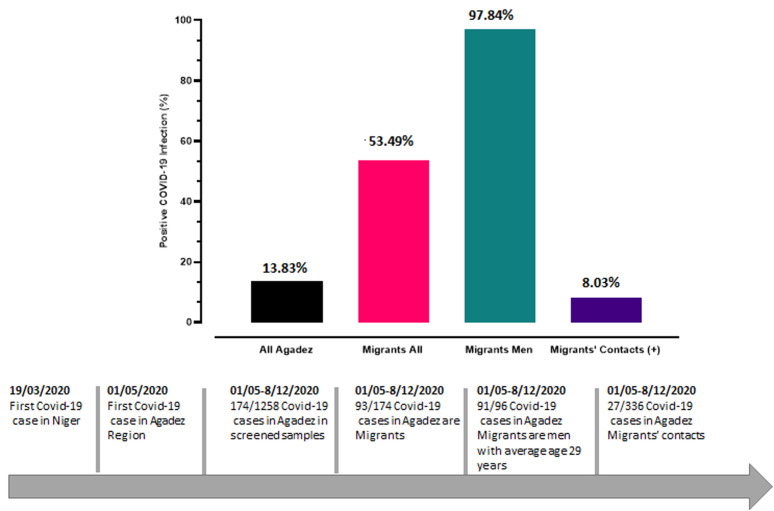
chronology of COVID-19 and characteristic of migrants infected with COVID-19 infection in Agadez Region in Niger from 01/05/2020 to 08/12/2020

Notwithstanding the mobility limitations accompanying the COVID-19 pandemic restriction, migrants continue to embark on clandestine journeys, escaping conflict and hardship in search of a more stable life. Although many deaths attributable to COVID-19 infection among migrants' workers have been underreported, it has been estimated that over 2,500 migrants have died during migration in 2020, as measures to contain the pandemic, such as lockdowns and travel restrictions, have increased the risks and uncertainties associated with these journeys. Numerous fatalities among migrant workers associated with COVID-19 infection and related measures were not included in this figure. [1]. The Agadez Region in Niger is experiencing the consequences of destabilization in the Sahel by armed groups while being the gateway to Europe for most Africans. Given its size (half of the country land), Agadez is a major challenge for epidemiological surveillance. As migration is a contributing factor to the spread of COVID-19 disease [2], we hypothesized that forcibly displaced persons are at increased risk for COVID-19 infection in Agadez. In a retrospective study of the health data of the national health information system (supplemented as needed by consultation registers), we reviewed all COVID-19 positive migrant patient records admitted from 1^st^ May to 8^th^ December 2020. Of note, medical checks were performed at seven entrance gates on all incoming passengers by trained health workers. A team of 58 officers with different backgrounds under the direction of the referring physician managed confirmed cases.

For holistic approach, two psychologists recruited by WHO provided psycho-social support to COVID-19 patients and relatives. We used deidentified data and ensure principles of ethics are respected during data cleaning process and analysis. Graphpad prism V.8 was used for graphing results and for descriptive analysis of proportions. We found that between 1^st^ May 2020 date of notification of the first case of the region to 8^th^ December 2020, Agadez recorded 174 confirmed cases (age median/range: 29.3/ 6-75 years) of COVID-19 out of 1,258 samples taken (846 routine screenings). Of the 174, 93 (53.5%) were migrants (91 or 97.8% males) (Figure 1). Geographically, of the 93 positive migrant cases, 91 cases were recorded in common Agadez and two in Assamaka. A successfully contact-tracing and testing was reported as 27/336 people contacts for the 93 positive migrants had also tested positive for COVID-19 infection. Among the 93 positive migrants, more than half were unemployed. Positive cases among migrants were all asymptomatic and, all successfully recovered from COVID-19 infection and benefited with psychologic support. Depression was common among migrants infected for SARS-CoV-2 in and related with the forced return from Algeria and confirmed COVID-19-infection.

The deadliest immigration path in the world remains the central Mediterranean-some 580 people perished on this route between March and October 2020. Hereby, we provide an earlier insight of COVID-19 infection among migrants in Agadez Region, Niger. We found that migrants are more likely of being young [1,2] and at increased risk for SARS-COV-2 infection [2]. In addition to language as barrier to quality of care access in the emergence room [3], mental health has been of concern among migrants worldwide [4-6]. The continuous arrival of migrant in this region makes hard contact tracing of positive cases. Moreover, considering the substantial positivity rate observed, the current over-whelmed site (Assamaka-Alit) might not be able of providing necessary infection and prevention controls measures such as observing physical distance. It is therefore a call for stakeholders involved in the region and for the African Union leadership to address the crisis and to consider migrants in the COVID-19 vaccination program.

Further, we found that migrants were more likely at working in informal sector as many of them could not fully describe their living job. Consequently, migrant involved in this precarious employment were less likely to be considered as essential workers but they work daily and cannot qualify for sick leave or for social security if any [7]. Effort is needed to address socio-economic challenges that face migrants in COVID-19 era. As such, communication in Agadez is more oriented towards migration management; above all, the peaceful cohabitation between migrants and indigenous peoples, in particular the management of the rumor that COVID-19 is being spread by migration. Despite being informative, the generalizability of our findings is limited by the fact that the reported high rate of positive cases among migrants in Agadez could be linked to the routine COVID-19 testing among migrants compared to residents due to lack of routine testing performed at the community level. For efficient border migration management in COVID-19 era; collaboration agreements and contingency plans for public health activities between countries and stakeholders are essential at common ports of entry, notably between Niger, Algeria and Libya. While a holistic approach is urgently needed to improve migrants´ health and social-economic conditions during COVID-19 pandemic, effort is needed to eradicate forcibly migration in Africa.

**Ethical approval and consent to participate:** this study used routine program data retrospectively collected with no individual data identifiers revealed or used. The study was approved by the Niger, Ministry of Public Health, and the Niger National COVID-19 Multisectoral Response Committee.
